# The interplay between DNA topoisomerase 2α post-translational modifications and drug resistance

**DOI:** 10.20517/cdr.2019.114

**Published:** 2020-02-27

**Authors:** Christophe Lotz, Valérie Lamour

**Affiliations:** ^1^Integrative Structural Biology Department, IGBMC, Université de Strasbourg, CNRS UMR 7104, INSERM U1258, Illkirch 67404, France.; ^2^Hôpitaux Universitaires de Strasbourg, Strasbourg 67000, France.

**Keywords:** DNA topoisomerase, drug resistance, post-translational modifications, etoposide

## Abstract

The type 2 DNA topoisomerases (Top2) are conserved enzymes and biomarkers for cell proliferation. The catalytic activities of the human isoform Top2α are essential for the regulation of DNA topology during DNA replication, transcription, and chromosome segregation. Top2α is a prominent target for anti-cancer drugs and is highly regulated by post-translational modifications (PTM). Despite an increasing number of proteomic studies, the extent of PTM in cancer cells and its importance in drug response remains largely uncharacterized. In this review, we highlight the different modifications affecting the human Top2α in healthy and cancer cells, taking advantage of the structure-function information accumulated in the past decades. We also overview the regulation of Top2α by PTM, the level of PTM in cancer cells, and the resistance to therapeutic compounds targeting the Top2 enzyme. Altogether, this review underlines the importance of future studies addressing more systematically the interplay between PTM and Top2 drug resistance.

## Introduction

The type 2 DNA topoisomerases (Top2) are evolutionary conserved enzymes and biomarkers for cell proliferation^[1]^. They are involved in essential cellular processes such as DNA replication, DNA transcription, and chromosome segregation^[2]^. The human Top2α isoform is highly expressed during mitosis and is essential for cell division^[3]^. Its main function is to regulate topological entanglements in DNA that can compromise cell division or gene transcription^[4]^.

**Figure 1:**
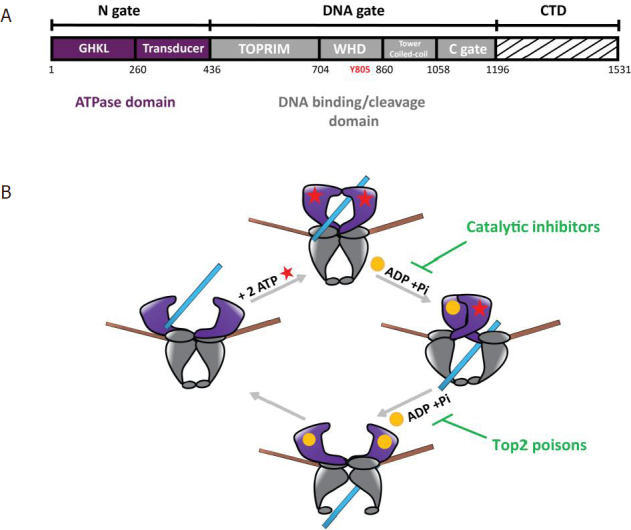
Domain organization of Type 2 DNA topoisomerase and catalytic cycle. A: domain organization of the human Top2 enzymes. The Top2 enzymes are composed of three dimeric interfaces. The N-gate (in purple) is composed of a GHKL domain binding ATP and a transducer domain. The DNA-gate (in grey) comprises the WHD with the conserved catalytic tyrosine (Y805 in red) and the Toprim domain, that contains three acidic residues binding magnesium ions. The C-gate forms a dimeric interface where the DNA exits the enzyme at the end of the reaction. The CTD (dashed lines) differs between species and between the human isoforms Top2α and Top2β. Residue numbering is that of the Top2α isoform. B: catalytic cycle of Top2 relaxation activity. (1) The Top2 enzyme binds a DNA G-segment (in brown) at the dimeric interface at the DNA-gate. (2) Upon ATP binding (red stars), a second DNA fragment called T-segment (in blue) is trapped in the ATPase domain. (3) Introduction of a reversible DNA double strand break in the G-segment is coupled to the hydrolysis of an ATP molecule, leading to the release of ADP (yellow sphere) and an inorganic phosphate, and results in the transport of the T-segment through the break. Catalytic inhibitors such as bispiperazine (ICRF derivatives) trap a closed clamp intermediate, affecting ATP-driven conformational changes of the allosteric assembly. (4) Hydrolysis of a second ATP molecule triggers the release of the T-segment through the C-gate and resets the enzyme. Top2 poisons such as etoposide and doxorubicin prevent G-segment religation. Top2: type 2 DNA topoisomerase; CTD: C-terminal domain; WHD: winged helix domain; G: gated; T: transported; GHKL: Gyrase, Hsp90, Histidine Kinase, MutL domain; TOPRIM: topoisomerase-primase domain

Top2 are large multidomain enzymes that fold into a homodimer forming three-protein interfaces called “gates”^[5]^ [Figure 1A]. The C-terminal domain (CTD) whose 3D structure is unknown is less conserved and seems to play a role in protein-DNA and protein-protein interactions^[6]^. To remove topological stress, Top2 introduce a reversible double strand break in a DNA molecule and transport another DNA duplex through the break^[5]^ [Figure 1B]. This elaborate mechanism can be targeted by chemicals that affect the catalytic sites. In particular, the Top2α isoform is a major target for antineoplastic agents that are widely used in cancer chemotherapy^[7]^. While catalytic inhibitors affect ATP hydrolysis, topoisomerase “poisons” stabilize the cleavage complex (Top2cc), leading to accumulation of lethal DNA double-strand breaks^[8,9]^ [Figure 1B]. However, cancer cells may develop resistance that can be attributed to Top2 single point mutations, alteration of gene expression, or regulation of post-translational modifications (PTM)^[10]^.

Aberrant PTM are often found in cancer cells and are one of their distinguishing features^[11,12]^. The earliest studies of PTM in Top2 proteins date back to the 1990s with the first report of a phosphorylation of purified Top2α from mouse cells^[13]^. Since then, the identification and characterization of PTM have been accelerated by the development of proteomic techniques^[14]^. Because of their relevance in cancer therapy, Top2α PTM have been characterized mostly in cancer cell lines, while the extent of PTM in the homeostasis of normal cells has been somewhat neglected^[15]^. Recently, we identified the phosphorylation and acetylation sites in human Top2α overproduced in the yeast *S. cerevisiae* and in a hamster mammalian cell line (BHK21), thus providing a basal landscape of the modifications for Top2α isoform^[16]^.

In this review, we analyze the different types of modifications affecting the human Top2α in normal and cancer cells with a structure-function perspective. We also overview the relationships among the regulation of Top2α by PTM, the level of PTM in cancer cells, and the resistance to anti-Top2 therapeutic compounds.

## Phosphorylations

Phosphorylation is the most studied modification of Top2 due to its importance in the regulation of the enzyme during the cell cycle and was shown to influence several aspects of Top2α function. The level of phosphorylation reaches a maximum peak at G2/M phase along with the level of Top2α expression^[17]^. Several studies have reported that Top2α catalytic activity is modulated by phosphorylations^[18,19]^.

**Figure 2:**
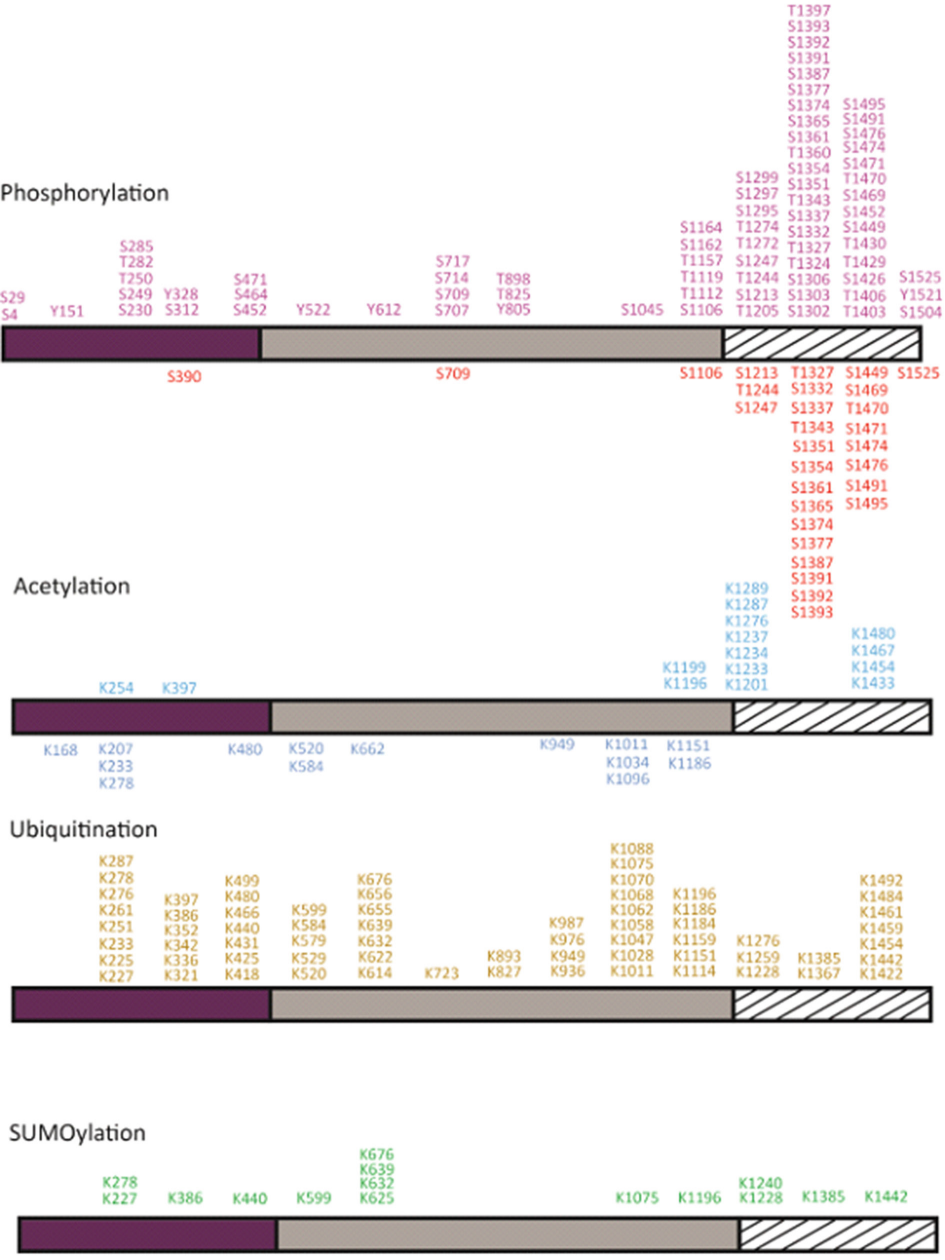
Post-translational modifications of the human Top2α. Phosphorylations and acetylations identified in the recombinant Top2α protein produced in yeast or mammalian cells are reported below the domain diagram (Bedez *et al*.^[16]^, 2018). Modifications identified in cancer cells as reported in the PhosphoSiteplus database are indicated above the domain diagrams (Horneck *et al*.^[40]^, 2014). Phosphorylation sites are colored in red, acetylation in blue, ubiquitination in yellow, and SUMOylation in green. To date, about 104 phosphorylation, 28 acetylation, 67 ubiquitination, and 15 SUMOylation sites have been identified. Twenty-six out of 29 phosphorylations in normal cells and 46 out of 75 phosphorylations in cancer cells were found in the CTD. Fifteen SUMOylation sites were identified in cancer cells, half on the DNA gate and C-gate, four in the N-gate, and five in the CTD. Thirteen acetylations on the CTD and two in the ATPase domain were reported in cancer cells. Two phosphotyrosines were identified in the N-gate, three in the DNA-gate, and one on the CTD in cancer cells. Positions are numbered as indicated in the NCBI gene ID:7153, which results in a +1 shift in the residue numbering for some positions compared with their numbering in articles (for example, Thr1342 appears in the present figure as Thr1343, and Ser1524 as Ser1525). Top2: type 2 DNA topoisomerase; CTD: C-terminal domain

The majority of phosphorylations were identified in the Top2α CTD in normal and cancer cells [Figure 2]. The CTD is important for the proper subcellular localization of the enzyme as its deletion leads to Top2 mislocalization^[20]^. In particular, the phosphorylation of Top2α Ser1213 is important for its relocalization from the chromosome arms to the centromere^[21]^. Phosphorylation by Casein Kinase II (CKII), Protein Kinase C (PKC), or Extracellular signal Regulated Kinase II enhanced the decatenation and the relaxation activities, of both human and *Drosophila* Top2α^[17,19,22]^. Inversely, incubation of Top2α with the kinase PKCζ inhibits the decatenation activity *in vivo* and *in vitro*^[23]^. Phosphorylation of Ser1106 by Casein Kinase I was shown to regulate decatenation since its mutation to alanine slows down the decatenation and cleavage reactions^[18,24]^. However, the presence of a phosphorylated residue cannot always be directly correlated with the modulation of the enzyme activity^[25]^. Substitution of Ser1525 with an alanine does not affect decatenation activity, despite being a major substrate for phosphorylation by CKII, Polo-like kinase I, and p38g^[26-29]^.

In addition to the regulation of Top2α catalytic activity, phosphorylations appear to be important for the recruitment of protein partners in a chromatin context. In *HeLa* cells, the methylated tail of Histone 3 was shown to interact with the Top2α chromatin tether, a 30-amino-acid sequence at the end of the CTD^[30]^. In this region, three phosphorylations could be identified in cancer cells and only one thus far in normal cells, including phosphorylation on Ser1525, which could modulate binding of Top2α to the nucleosome [Figure 2].

**Figure 3:**
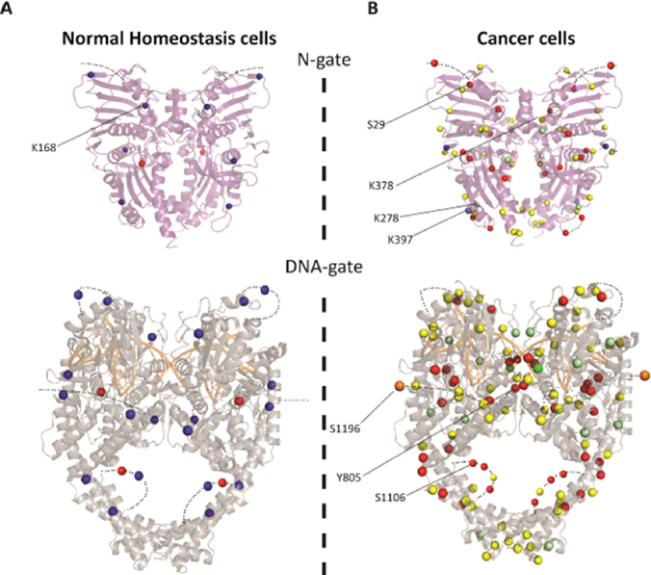
Distribution of the post-translational modifications on the structures of Top2α catalytic domains. A: modifications found in normal homeostasis cells (Bedez *et al*.^[16]^, 2018) reported on the ATPase domain structure (PDBID: 1ZXM) (top) and DNA binding/cleavage domain (PDBID: 5GWK) (bottom). B: modifications found in cancer cells reported in the PhosphoSitePlus database reported on the catalytic domains. The C-terminal domains for which no structure is available are not represented. Modifications appear as a blue sphere for acetylation, red for phosphorylation and yellow for ubiquitination. Positions that were found either ubiquitinated or SUMOylated appear as pale green spheres. Positions that were found either ubiquitinated or acetylated appear as pale blue spheres. Half of the SUMOylations were identified in the TOPRIM domain and C-gate. Only one position at residue 625 was found solely SUMOylated (dark green) and residue 1196 was found to be the site of acetylation, SUMOylation, and ubiquitination (orange). Positions specifically mentioned in the main text are indicated by a black line. Figures were generated using PyMOL Molecular Graphics System, Version 2.0 Schrödinger, LLC. TOPRIM: topoisomerase-primase domain

In the conserved catalytic domains of Top2α, three phosphorylation sites in the ATPase, topoisomerase-primase domain (TOPRIM), and the coiled-coil domains were detected in normal cells [Figure 3]. Ten additional phosphorylations were identified in cancer cells in the GHKL and transducer domains and in the N-terminal arm closing the dimer [Figure 3]. Phosphorylation of Ser29 by PKC in the N-terminal arm of the ATPase domain stimulates the DNA relaxation activity *in vitro*^[17]^. Phosphorylations at this position could impact the allosteric movements of the ATPase domain and the dimeric interface.

The overall level of phosphorylation was shown to modulate the ATPase activity of Top2α as well as the cleavage/religation reaction *in vitro*^[22,31,32]^. More phosphorylations can be found in cancer cells in the TOPRIM and winged helix domain (WHD) domains, and lining the coiled-coil region [Figures 2 and 3B]. Although less abundant, six phosphotyrosines in the Top2α protein were identified in cancer cells [Figure 3B].

Interestingly, the conserved catalytic tyrosine (Y805) was found to be phosphorylated in acute T-cell leukemia (Jurkat cells) and myelogenous leukemia K562 cells, and not in normal cells, suggesting direct modulation of the enzyme cleavage activity.

The phosphorylation status of Top2 in drug resistant cells has been the subject of contradictory observations. Takano *et al*.^[33]^ observed that hyperphosphorylation of Top2 in etoposide-resistant KB cells correlated with a decrease in cleavage complex formation. CKII is hyperactivated in cancer while in fission yeast CKII phosphorylation of Top2 suppresses its sensitivity to the catalytic inhibitor ICRF-193, which inhibits Top2 by impairing its ATP hydrolysis activity^[34]^. In this context, phosphorylation of serine residues was identified in the CTD outside the ATPase domain. This indicates not only that distant positions can have a role in the response to ICRF, but also that the phosphorylation status of Top2 can affect its drug response. Hyperphosphorylation linked to CKII was also observed in drug resistant breast cancer cells^[35]^. In contrast, hypophosphorylated Top2α was observed in etoposide-resistant K562 and HL-60 leukemia cells, being in the latter case linked to a reduced level of Ser1106 phosphorylation^[36,37]^.

## Acetylations

We identified acetylation sites almost exclusively in the structured catalytic core of Top2α and only one in the CTD after overexpression in yeast and BHK21 cells^[16]^. This is consistent with the observation that acetylations are often found in structured regions of proteins^[38]^. In cancer cells, acetylations were mostly identified in the CTD^[39-41]^ [Figure 2]. Acetylations have not been studied as extensively as phosphorylations, which limits the conclusions that can be drawn from a comparison between normal homeostasis and cancer cells.

In healthy cells, the acetylations found in the DNA gate are located in the TOPRIM, coiled-coil, and C-gate domains. Those located close to the extremities of the DNA groove could affect DNA binding [Figure 3A]. Interestingly, 8 acetylated positions in healthy cells located in the DNA gate have also been found ubiquitinylated in cancer cell lines, suggesting a switch of modification in cancer cells^[42-46]^. Lys1196 was found to be a site for acetylation in Jurkat cells, ubiquitination in Jurkat and HEP-2 cells, and SUMOylation in *HeLa* cells^[43,46,47]^. This residue is located in a hinge region between the C-gate domain and the CTD and could be involved in the regulation of Top2 activities [Figure 3B]. Lys278 in a loop region of the transducer is acetylated in normal cells but found to be SUMOylated and ubiquitinylated in cancer cells [Figure 2]. Lys397, which lies in the transducer helix, is acetylated in cancer cells but can also be ubiquitinated. These positions are mostly located on the surface of the N-gate domain and therefore compatible with the addition of bulky modifications [Figure 3A]. Although buried at the dimeric interface, we also found an acetylation on Lys168 in Top2α produced in the yeast expression system. Mutation of this residue showed that this position is important for the coupling of ATPase activity and dimerization^[16]^.

While few studies are available on the acetylation of Top2α in response to drugs, indirect inferences can be made. The association of Top2 with histone deacetylases (HDAC) was shown to modulate its activity and in particular etoposide-stimulated cleavage both *in vivo* and *in vitro*^[48,49]^. In addition, treatment of hepatocellular carcinoma cells with an HDAC inhibitor triggers the proteasomal degradation of Top2^[50]^.

Interestingly, acetylations that were identified in BHK21 were also found as targets for ubiquitination in cancer cells. Inversely, most acetylations identified thus far in cancer cells do not correspond to reported ubiquitination or SUMOylation sites, which indicates that regulation of some PTM depends on the cell state. Although further analysis is needed, these studies suggest a regulation of Top2α activity by acetylations and a potential interplay between other modifications such as ubiquitinations and SUMOylations.

## Ubiquitinations and sumoylations

Ubiquitin is a small regulatory protein that when attached to proteins alter their cellular localization, protein activity, or molecular interactions, and may target them for proteasome degradation^[51]^. Ubiquitinations were identified throughout the Top2α sequence in cancer cells [Figure 2]. In non-cancer cells (mouse embryonic fibroblasts), only one ubiquitination in the ATPase transducer domain has been reported thus far. Lys378 is ubiquitinated by the E3 ligase activity of the APC/C complex, promoting 26S proteasome degradation at G1 phase, thus modulating Top2α levels for chromosome maintenance^[52]^. This residue interacts directly with the ATP molecule and could be accessible to modifications when the N-gate is open during the catalytic cycle.

Introduction or removal of ubiquitin is also a mechanism of drug resistance, as it modulates the Top2α activities and protein levels through proteasome degradation^[53-55]^. Deficiency in the RNF168 E3 ubiquitin ligase in breast cancer cells, or elevated levels of ubiquitin ligase Mdm2 in osteosarcoma cells, confers resistance to the Top2 poison etoposide, by regulating Top2 activities^[53,56]^_._ Ubiquitin-mediated degradation of Top2 also contributes to the level of drug resistance in solid tumors since proteasome inhibition leads to etoposide resistance^[57]^. In addition, the E3 ubiquitin ligase Bmi1/Ring1A controls the proteasomal degradation of Top2cc in *HeLa* cells upon teniposide treatment^[58]^.

Evidence for a functional and physical interplay between ubiquitination and SUMOylation have been reported at a larger scale, suggesting a coordination for proteasome degradation and the regulation of ubiquitin modifiers^[59]^. The Small Ubiquitin-like Modifier or SUMO proteins are considered as members of the ubiquitin-like protein family, although they are not directly related to protein degradation^[60]^. It was shown that the resolution of TOP2cc by tyrosyl-DNA phosphoesterase 2 is controlled by the SUMO ligase ZATT^[61]^. SUMOylation play a critical role in DNA condensation and chromosome segregation. Top2α is directed to the inner centromere via the E3 ligase RanBP2-mediated SUMOylation for the resolution of sister centromeres^[62]^. The E3 ligase PIASγ was also shown to regulate the catalytic activity of Top2α at the centromere for the proper segregation of chromosome^[63]^. Evidence of a crosstalk between phosphorylation and SUMOylation was shown in cancer cells, which targets the CTD^[64,65]^.

Strikingly, most of the SUMO sites in the catalytic domains were identified along the dimeric interface of the Top2α structure [Figure 3]. Structure determination of Top2 has shown that the subunits form an intertwined dimer structure and that the buried surface could be accessible during the catalytic cycle [Figure 1B]^[66-69]^.

SUMOylation, similar to other post-translational modifications, is impacted by chemical adjuvants but little information is available thus far. Conjugation to the SUMO2/3 by the SUMO ligase PIASg in response to Top2 inhibitors alters the Top2 decatenation activity, essential for chromatid arm separation at mitosis^[70]^. Interestingly, SUMOylation in Top2α is increased upon ICRF-193 or teniposide exposure, as well as following oxidative stress or heat shock^[71]^.

Altogether, ubiquitination and SUMOylation are important modifications that can have a direct impact on Top2α levels, interplay with other PTM, and consequently affect drug response.

## Top2α mutations in resistant cells

**Figure 4:**
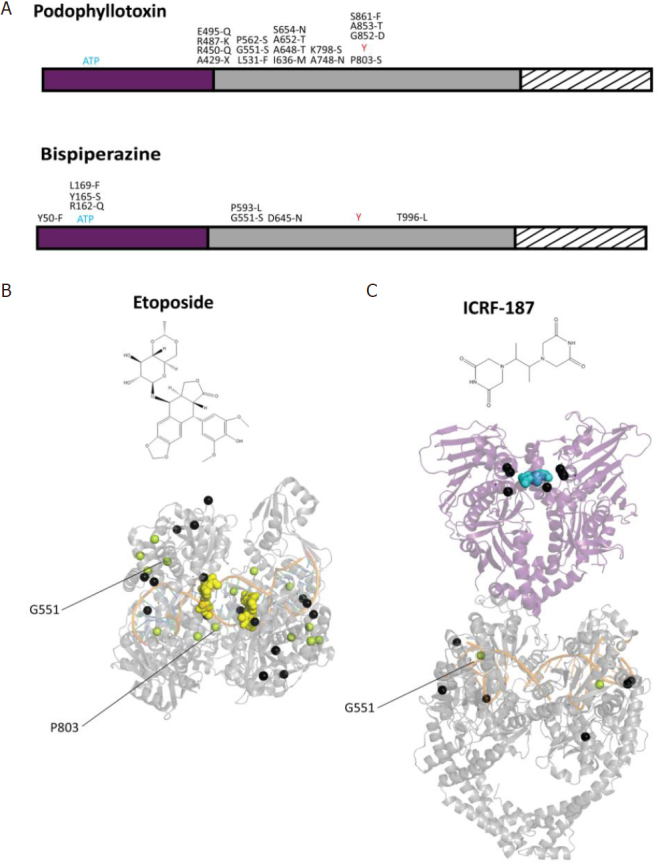
Distribution of point mutations providing drug resistance on the structures of the Top2α catalytic domains. A: distribution of the positions presenting single point mutations that confer resistance to podophyllotoxin compounds (etoposide) (top) and bispiperazine (ICRF-187) (bottom), indicated on the domain diagram of Top2α. B: single point mutations conferring resistance to anti-Top2 drugs are represented as black spheres reported on the structures of the catalytic domains. The DNA binding/cleavage domain (PDBID: 5GWK) of human Top2α homolog bound to two etoposide molecules represented as yellow spheres (B). The ATPase domain of the yeast homolog (PDBID: 1QZR) bound to the ICRF-187 compound represented as blue spheres, with mutations also appearing in the DNA binding/cleavage domain (PDBID: 5GWK) (C). Residues mutated to serine in resistant cell lines appear in pale green. Positions specifically mentioned in the main text are indicated by a black line

Point mutations in the Top2α gene that lead to podophyllotoxin resistance mostly affect the drug binding site or are located on the TOPRIM domain [Figure 4A]. A recent study in yeast identified mutations of Top2 conferring resistance against vosaroxin, a quinolone derivative in a phase II clinical trial^[72]^. Some mutations also appeared in the unstructured C-terminal region of human Top2α, indicating that residues that are external to the binding pockets of the drugs can contribute to resistance mechanisms, due to the allosteric properties of the protein.

Point mutations conferring resistance to the bispiperazine compounds were found in the ATPase domain of Top2α in small cell lung cancer and Chinese hamster ovary cells^[73]^. These mutations impact the dimeric interface and the formation of the ICRF binding pocket^[74]^ [Figure 4]. Another study in yeast showed that drug resistance mutations are not restricted to the N-terminal domain but can also be found in the DNA-gate^[75]^ [Figure 4B]. Single point mutations located in the N-terminal domain display a more resistant phenotype compared with those in the DNA-gate, with the exception of Gly551, a conserved residue in eukaryotic Top2. Interestingly, a Gly551Ser mutation confers resistance to both Top2 poison etoposide and the catalytic inhibitor ICRF^[72,75]^. The dual resistance could be explained by the proximity of the etoposide-binding site in the DNA groove and the allosteric properties of the enzyme, since movements of the DNA-gate are coupled to ATP hydrolysis [Figure 1B].

Although no drug resistant mutations targeting known PTM sites could be found in the literature except for vosaroxin resistant yeast cells^[72]^, it cannot be excluded that such events occur in the Top2α gene of resistant cancer cells. Mutations have been reported that introduce a serine or threonine in etoposide-resistant cells in the TOPRIM or WHD domains^[73,75]^ [Figure 4]. *In silico* prediction of phosphorylation sites indicates that mutation of Ala652, located between TOPRIM and WHD, would generate a phosphorylation site for PKC^[76]^. Mutation of Pro803 nearby the catalytic tyrosine shows a consensus sequence for Cdc2. Further investigations would be required to find if these positions are targeted by kinases *in vivo* and contribute to the mechanism of resistance.

## Conclusion

Most phosphorylations were found in the CTD of the Top2α isoform, related to its role in the cell cycle. However, the conserved catalytic domains are also directly targeted by kinases and other modifying enzymes introducing acetylations, SUMOylations, and ubiquitinations, interfering with the structure-function properties of the Top2α isoform. Top2 PTM in cancer cells were mostly identified in targeted studies analyzing their regulation and interactions with modifying enzymes during the cell cycle. Further systematic analysis of Top2 PTM in cancer cells would be required to analyze the interplay between PTM and compare their modulation in response to different compounds, in order to identify potential biomarkers of cancer prognosis and drug resistance, as well as new therapeutic avenues targeting the Top2α or modifying enzymes.

## Declarations

### Copyright

© The Author(s) 2020.
